# Editorial Correspondence

**Published:** 1860

**Authors:** W. F. Westmoreland

**Affiliations:** Philadelphia


### EDITORIAL AND MISCELLANEOUS.

### Editorial Correspondence.

PHILADELPHIA, Jan. 1860.

DR. JOSEPH P. LOGAN—

*Dear Sir:* I arrived in Philadelphia several days ago, but have been so much engaged that I have had no time, until now, to redeem a promise made before leaving, to drop you an occasional note in regard to matters and things medical, in the two great rival cities.

The exciting topic in medical circles, here and elsewhere that I have stopped, is the exodus of Southern Medical Students from Northern Colleges. I do not propose here to discuss the propriety of this movement, as all, however rabid, are forced to admit that the young gentlemen had a perfect right to act just as they pleased in the matter. Had less been said, and more temperate language used against the movement and those engaged in the secession, it would have been far better for those who will likely feel it most. From all I can learn, there were between two hundred and fifty and three hundred who left Philadelphia—four-fifths, or perhaps more, of this number, were matriculates of the Jefferson Medical College.

Notwithstanding the exodus, both the leading schools still have large classess—sufficiently large, in all reason, to satisfy any ordinary ambition. The Jefferson Medical College numbers, the present course, 630 Matriculates, and the University of Pennsylvania the rise of 500—the largest classes, I believe, ever in attendance in Philadelphia.

My first day in the "City of Brotherly Love," was spent at the Jefferson Medical College (my Alma Mater). This visit was opportune, as it was the day for Clinical instruction. I had the pleasure here of meeting the two distinguished Professors in charge of the Clinic, Drs. Pancoast and Dunglison, to whom I am under lasting obligations for the many courtesies extended me during my stay in the city.—Prof. Dunglison conducted the medical Clinic and presented quite a number of interesting cases. Although a half score or more years have elapsed since I have had the pleasure of meeting the Doctor in the lecture room, I am happy to say that time has made but little change in his personal appearance, and, mentally, he has lost none of his brilliancy—he is the same learned and elequent teacher. The Surgical Clinic was conducted by Dr. Pancoast, one of America's greatest surgeons. He had ample material at his command, and presented quite a variety of surgical disease. At a subsequent Clinic, I saw him operate for that troublesome affection, Lachrymal fistula. He performed the operation which he suggested some years ago, and since practiced, he says, with great success. The peculiarity of the operation consists, first, in a section of the stricture or strictures of the duct, which he does by means of a bistoury with a blade sufficiently narrow to pass into the osseous canal, thus making the section of the constricted portion of the duct as the blade is introduced into the canal. He next introduces into the duct an ivory tube from which the earthy matter has been removed by maceration in dilute muriatic acid. The tube is introduced, and the external wound closed, as in Dupuytrens, with the metallic tubes. He contends that the tube is very soon imbued with the moisture of the sac and duct, enlarges and becomes flexible, and is less irritating than any substance yet suggested to dilate the duct. In a few months the tube is removed by disintegration and absorption, and the patient permanently relieved. The theory is plausible, and it is to be hoped that it will stand the test of experience.

The Clinic of the Jefferson Medical College is certainly the best in Philadelphia, and constitutes all the instruction in that department that is worth much to the students composing the class. It is true that they have in the city two large

Hospitals, the Pennsylnania and Blockley, with any number of patients, but so far as the masses of students are concerned, they had just as well be in an adjoining State. I will venture the assertion that not one student in ten, from this or other Colleges in the city, will visit the hospitals half a dozen times during the present session. And if they did, they would have no more advantages, nor would they see or learn more than they would at the College Clinics. At the Pennsylvania Hospital they have precisely the same kind of instruction. The patients brought into the amphitheatre, examined and prescribed for in the presence of the class. It is true that there are some who have positions in the wards, or who are the private students of those who have access to the Hospitals, that are, or at least may be, greatly benefitted.

Through the courtesy of Dr. Pancoast, a son of the distinguished Prof. of Anatomy, a day or two ago, I had the pleasure of a visit to the Pennsylvania Hospital. I here met Dr. Hodge, Jr., resident Surgeon in this great Charity, and to whom I am due many thanks for attentions shown me during this visit. My time was spent principally in the surgical wards, where I saw quite a number of interesting cases. The internal arrangement of this Hospital will compare favorably with the great European Charities. The same day I spent a pleasant hour at the University of Pennsylvania. Heard a very interesting lecture by the eminent Professor of Surgery in this institution, Dr. Henry H. Smith. I did not have an opportunity of making his acquaintance, which I very much regretted, but will certainly do so before leaving the city.

To-day I made the acquaintance of that distinguished Surgeon and author, Dr. Samuel D. Gross, Prof. of Surgery in the Jefferson Medical College. Dr. Gross has no superior in America as a teacher. I visit Blockley Hospital to-morrow, with the Doctor, to witness a resection of the knee-joint, the particulars of which I will give you in my next letter.

Very Respectfully,

Your Friend,

W. F. WESTMORELAND.

